# Echocardiographic measurements and cardiac anatomy in healthy Western hognose snakes (*Heterodon nasicus*)

**DOI:** 10.17221/63/2022-VETMED

**Published:** 2023-02-23

**Authors:** Matteo Oliveri, Cristina Carnabuci, Massimo Vignoli, Simone Di Feliciantonio, Marco Di Feliciantonio, Leonardo Della Salda, Zdenek Knotek, Morena di Tommaso, Alessia Luciani

**Affiliations:** ^1^Faculty of Veterinary Medicine, Teaching Veterinary Hospital, University of Teramo, Teramo, Italy; ^2^Avian and Exotic Animal Clinic, Faculty of Veterinary Medicine, University of Veterinary Sciences Brno, Brno, Czech Republic

**Keywords:** 2D mode, cardiology, doppler, echo Doppler, heart, M-mode, physiology

## Abstract

This study aims to describe the most important cardiac structures in the *Heterodon nasicus* through echocardiography and anatomical dissection. Echocardiographic and echo-Doppler measurements were performed on twenty healthy adult *Heterodon nasicus* (10.10). The values of the ventricular length, aortic diameter, pulmonary trunk diameter, the mean thickness of the interventricular septum, and thicknesses of the wall of the *cavum pulmonale* (Cav. P) and *cavum arteriosum* (Cav. A), were measured. The aortic flow and pulmonary trunk flow were recorded. Two dead specimens (1.1) were dissected. The male’s pulmonary trunk diameter was bigger compared to the female’s in both the long and short axis. The reproductive ecology of *Heterodon nasicus* has yet to be fully elucidated upon, however, male territorialism and dispersal from the hibernacula, and multiple male courtships toward a single female were described, hence, the more active reproductive activity of the male and the consequent sexual selection toward a higher aerobic performance can be hypothesised. A moderate interventricular right to left shunt was noticed in the Cav. V of all the specimens, which is considered normal and should not confuse the clinician. Congenital defects, cardiomyopathies, valvulopathies, and pericardial diseases are known to occur in ophidians and other reptiles. Reliable data and profound knowledge of the anatomy and physiology of the ophidian heart are fundamental for the *in vivo* diagnosis of cardiac diseases in snakes.

*Heterodon nasicus* is a colubrid snake belonging to the Dipsadinae subfamily. Due to its small size and tame temperament, *H.* *nasicus* is currently one of the most commonly kept colubrid snakes in Europe. Snakes are known to suffer from a number of cardiac illnesses such as myocarditis, endocarditis, infarcts, pericarditis, parasitic infestation, and valvular insufficiencies (Barten and Frye 1981; Schilliger et al. 2010a; Schilliger et al. 2016). Despite the growing amount of data collected in the last few decades, publications dealing with the antemortem diagnosis of cardiac diseases in reptiles and reports of their corresponding treatments are still scarce, and most of the aforementioned illnesses are diagnosed post-mortem.

Ultrasonography and, to a lesser extent, echocardiography are well established, non-invasive diagnostic methods in reptile medicine (Schilliger et al. 2010b). Echocardiography has proven to be a suitable method for the early diagnosis of cardiac illnesses in snakes (Schilliger et al. 2010a; Schilliger et al. 2010b), however, to enhance the effectiveness of this technique and its applicability in clinical practice, more accurate species-specific data is needed.

Therefore, this study aims to describe a suitable method for echocardiography in the Western hognose snake (*Heterodon nasicus*), to describe the most important structures, and to describe the heart through echography and anatomical dissection. A detailed comprehension of ophidian cardiac anatomy, and reliable data are necessary for the ante-mortem diagnosis of cardiac illnesses in snakes and will be extremely useful to the clinician attempting cardiological exploration in *Heterodon nasicus*.

## MATERIAL AND METHODS

Echocardiography was performed during a routine health assessment before hibernation in twenty (10.10) adult Western hognose snakes (*Heterodon nasicus*). The male snakes ranged from 60 g to 133 g (mean 83.4 ± 22 g); the females of this species are notoriously larger and ranged from 180 g to 300 g (mean 230 ± 39.3 g). All the specimens were three years of age by the time of the experiment. All the subjects were captive-bred and housed individually. The cage sizes for the females and males were 40 × 56 × 15 cm and 45 × 20 × 10 cm, respectively. The cages were heated with heat panels for 1/3 of the area. The temperature in the hot spot was 31 °C to 32 °C, lowering to 22 °C to 25 °C in the coolest part of the terraria. The females were fed every 7 days with 25 g to 30 g of mice (*Mus musculus*), and the males were fed every 10 days with 10 g to 15 g of mice. The humidity ranged between 60% and 75%. All the subjects were given a complete clinical examination. None of the animals showed any signs of illness. Postprandial myocardial hypertrophy has been described in snakes (Wang et al. 2003; Andersen et al. 2005; Riquelme et al. 2011; Enok et al. 2013). Therefore, the snakes were fasted for a week prior to the procedure. The temperature was recorded before and after the procedures using an infrared gun (Peak-meter infrared gun; Shenzhen Huayi Instrument Co., Guangdong, P.R. China). The snakes were manually restrained by a trained assistant, gently holding their heads behind the neck, and blocking the middle of the body. Any possible sign of distress was reported and noted, including attempts to bite, thanatosis, spontaneous oral bleeding (Fuentes et al. 2021), and discharge of the contents from the musk glands. The animals were examined by accessing the ventral scales (gastrosteges) and the lateral body wall. An abundant amount of gel was applied on the gastrosteges and the lateral body wall in correspondence with the heart.

**Figure 1:**
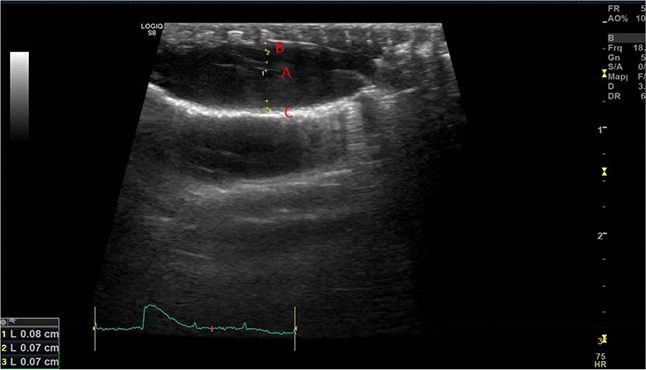
The long axis of the V from the ventral approach using the 2D method Measurement of the thickness of the IV septum (0.8 mm) “A”; the wall of the Cav. A (0.7 mm) “B”; and Cav. P (0.7 mm) “C”; were taken at end diastol

Two-dimensional (2D), M-mode, and Doppler echocardiographic examinations using ventral, and intercostal left and right approaches were performed by a single trained operator (C.C.). A Logiq S8 Vet ultrasonography machine (General Electric Healthcare, Boston, MA, USA) was used for the procedure. Each measurement was repeated three times, and the resulting mean value was recorded and used for the statistical analysis. The two-dimensional and M-mode echocardiography was performed with an 8–18 MHz phased array transducer (hockey stick micro-linear probe). The measurements of the total ventricular length, the thickness of the wall of the *cavum arteriosus* (Cav. A), of the interventricular (IV) septum, and of the *cavum pulmonale* (Cav. P) at the end of the diastole, were obtained from a ventral approach with a long axis (LA) view of the entire ventricle (V) and right atrium (Ra) using the 2D method. The total length of the V was measured at the end of the diastole, from the aortic arch to the apex. The *cavum venosum* (Cav. V) and Cav. P were measured with the leading edge to leading edge method (Lang et al. 2015), at the point of the maximum width (Figure 1).

**Figure 2:**
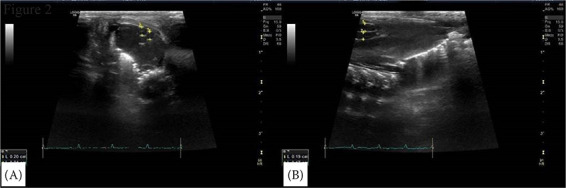
Aortic arches and pulmonary trunk in the SA (A) and LA (B) (A) The two aortic arches (2 mm) and pulmonary trunk diameters (2.1 mm) were examined and measured in the 2D mode. (B) The maximum diameters of the right aortic arch (1.7 mm) and the pulmonary trunk (1.9 mm) were measured in the M-mode. In this view, the two aortic arches are superimposed, allowing just one measurement

**Figure 3:**
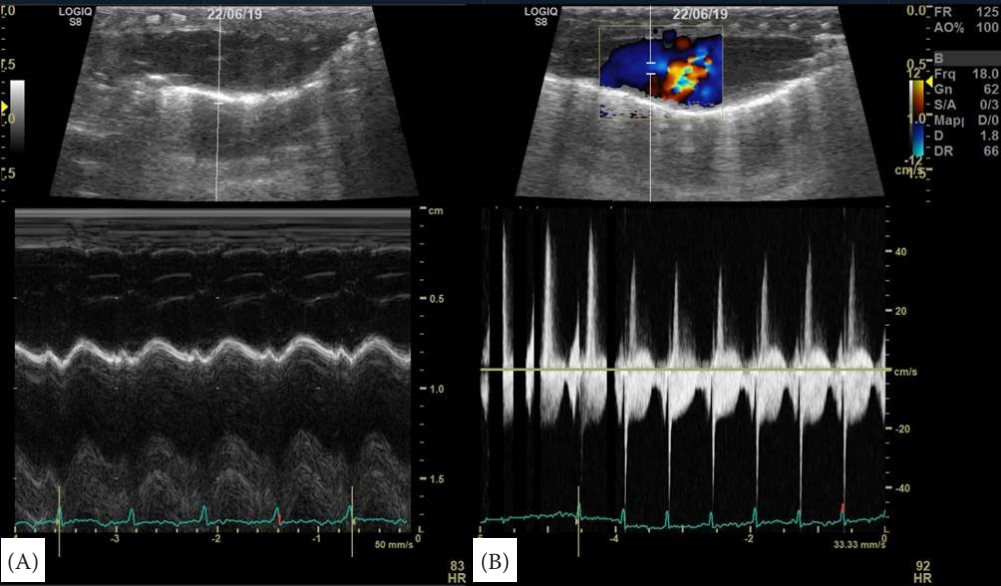
Image of the LA of the V in the B-mode (A), and echo-Doppler of the same chamber (B) In image (B), the pulsed wave Doppler was positioned at the emergence of the pulmonary trunk from a ventral approach, detecting a mean flow of 0.40 m/sec. Turbulent Doppler flow is visible caudal to the pulse wave Doppler and is consistent with the physiologic right-to-left shunt of the species at the level of the Cav. V

The long axis of the aortas and pulmonary trunk were evaluated from the intercostal (lateral) approach. From the right transarterial short axis (SA) view, the two aortic arches and pulmonary trunk diameters were examined and measured in the 2D mode, choosing the frame with the maximum diameter, using the leading edge to leading edge technique. With the same technique, from the long axis transarterial section, the maximum diameters of the right aortic arch and the pulmonary trunk were measured in the M-mode (Figure 2). The pulmonary and the aortic flow velocities were measured by positioning the pulsed wave Doppler at the emergence of the pulmonary trunk and the aortic arches from a ventral approach, and the maximum peak velocities were recorded. Due to the small size of the aortas, any differentiation between the right and left flows was impossible, and the flow was recorded as a single value (Figure 3).

**Figure 4:**
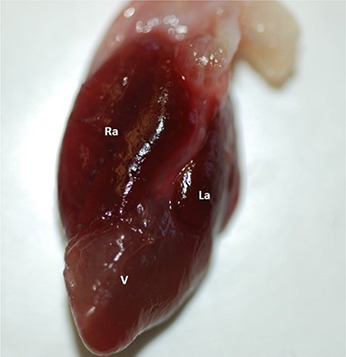
The dissected heart of a female *Heterodon nasicus*

**Figure 5:**
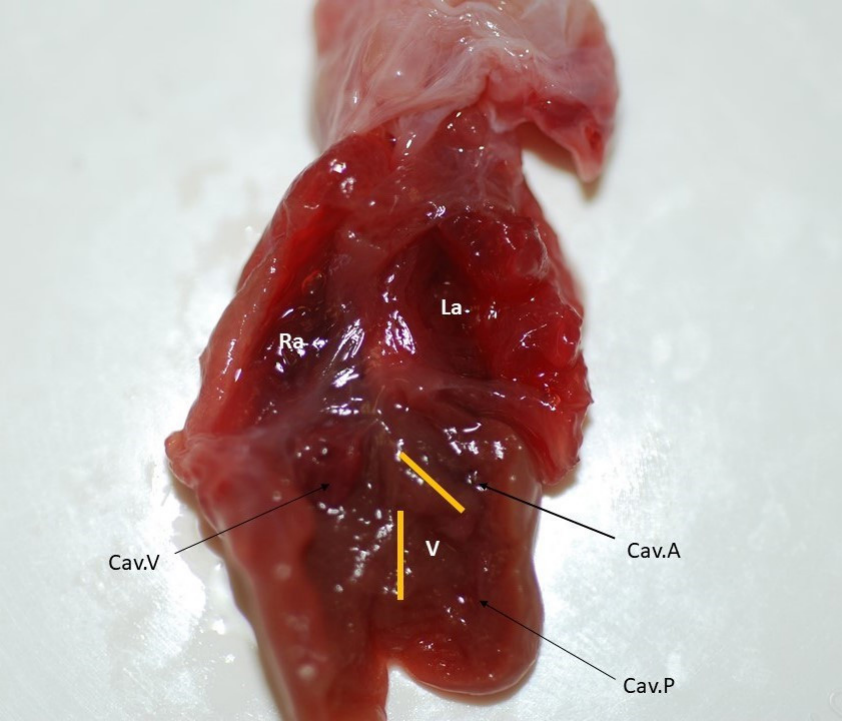
Heart of a female *Heterodon nasicus* – A longitudinal cut of the V and the atria exposed the main chambers of the V The yellow lines represent the IV septum (vertical septum), which rises from the apex of the V and travels upward, dividing the Cav. A, from the Cav. V and the muscular ridge (horizontal septum), which divides the Cav. V and Cav. P

**Figure 6:**
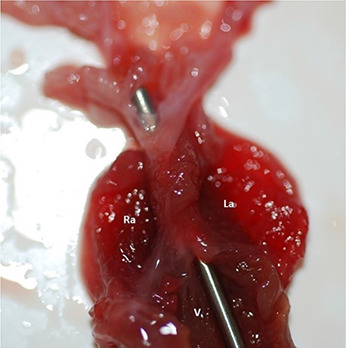
Heart of a female *Heterodon nasicus* – A metal probe was inserted in the pulmonary trunk to show the direction of the aortas and the pulmonary trunk

For a better understanding of the cardiac anatomy, two dead specimens, one adult male and one adult female, were dissected. The specimens originated from the same breeding facility and had died for unknown reasons. These two specimens did not belong to the same group of animals used for the experiment. During the necropsy, the gastrosteges over the heart were dissected using a ventrolateral approach. The pericardium was incised and the whole organ exposed. The major vessels were ligated and transected, and finally the heart was extracted. A transarterial cut on the long axis (LA) was performed in order to expose the inner structures of the organ, including the atria, the large vessels and the sub-chambers of the V (Figures 4–6).

A statistical analysis was performed using commercial software (GraphPad Prism v6.01 software; GraphPad Software Inc., San Diego, CA, USA). All the data were evaluated using a standard descriptive statistic and reported as the mean ± standard deviation (SD) or as the median and range (minimum–maximum), based on their distribution. Normality was checked by the D’Agostino Pearson test. According to Cornell et al. (2004), normalising the M-mode measurements through elevation of body weight to 1/3 provides the most reliable data, therefore, all the linear echocardiographic measurements and M-mode measurements were normalised to the body weight 1/3 (kg1/3) for each animal. The 2.5^th^ and 97.5^th^ percentiles were defined as the lower and upper reference limits, respectively. As recommended, 90% confidence intervals around these limits were also calculated following the guidelines of the American Association of Veterinary Clinical Pathology (Friedrichs et al. 2012). A comparison of the echocardiographic measurements between male and female was performed using the unpaired *t*-test or the Mann-Whitney test. The threshold of statistical significance was set at *P* < 0.05.

## RESULTS

The mean body temperature of the snakes before the procedures was 27.4 ± 0.5 °C and 28 ± 0.5 °C in the males and females, respectively. A significant rise in body temperature was noted in all the subjects (3.0 ± 1.1 °C in the males and 3.3 ± 0.7 °C in the females), likely due to muscular twitching and manipulation (Table 1).

**Table 1:** Characteristics of the snakes included in the study

No.	Sex	Weight (g)	Temp pre (°C)	Temp post (°C)	Temp rise (°C)
10	M	83.4 ± 22.0	27.4 ± 0.5	30.4 ± 0.8	3.0 ± 1.1
10	F	230.0 ± 39.3	28.0 ± 0.5	31.3 ± 1.1	3.3 ± 0.7

The body temperature was recorded before (Temp pre) and after (Temp post) the experiment. A significant rise in body temperature (Temp rise) was noted in all the subjects, likely due to muscular twitching and manipulation

F = female; M = male

The gross cardiac anatomy of *H. nasicus* is similar to that of other ophidians (Wyneken 2009). The heart is elongated, and lies within the coelom, surrounded by the pericardium and in close proximity to the cranial part of the right lung. It is a substantially asymmetric organ, with the right atrium (Ra) being larger than the left atrium (La) and extending slightly caudally (Figure 4). The macroscopic examination allowed the visualisation of the three major vessels leaving the V: the left and right aortas and the pulmonary trunk. A longitudinal cut of the V and the atria exposed the main chambers of the V, these are the Cav. V, Cav. A, and Cav. P (Figure 5)*.* Two septa were identified in the V; these structures define the very architecture of the V separating the chambers. The first and most prominent is the IV septum (vertical septum), which rises from the apex of the V and travels upward, dividing the Cav. A, from the Cav. V. The second smaller septum is the muscular ridge (horizontal septum), which divides the Cav. V and Cav. P (Figure 5). The aortas and the pulmonary trunk emerge from the V tightly connected to one another, and travel upward between the atria (Figure 6).

A manual restraint was suitable to adequately restrain the animals during the echocardiography. None of the subjects showed any sign of aggressiveness or discomfort during the whole procedure. The mean values and SD of the aortic diameter in SA and LA, and of the pulmonary trunk diameter in the SA and LA are presented in Table 2; the length of the V, the thickness of the IV septum (vertical septum) and the walls of the Cav. P and Cav. A are presented in Table 3. The recorded flows of the aortic arch and pulmonary trunk are presented in Table 4. Interestingly, the male specimens showed an increased *cavum arteriosum* and ventricle length compared to the females despite the clear disproportion in size, the data, however, are not statistically supported (Table 3). The pulmonary trunk diameter was bigger in the males compared to the females in both the long axis (*P* = 0.046 5) and short axis (*P* = 0.019 8). A moderate interventricular right to left shunt was noticed in the Cav. V of all the specimens (Figure 3).

**Table 2:** Intervals of the normalised M-mode measurements

Measurement	Sex	Mean	97.5 percentile	2.5 percentile	Upper 90% CL of mean	Lower 90% CL of mean	Std. error of mean	*P*-value
Diam aort SA (mm)	M	3.17 ± 0.39	3.59	2.51	3.39	2.94	0.12	0.366 8
	F	2.98 ± 0.51	3.70	2.24	3.28	2.68	0.16	
Diam aorta LA (mm)	M	3.14 ± 0.58	4.34	2.50	3.48	2.80	0.18	0.063 7
	F	2.65 ± 0.52	3.54	1.79	2.95	2.35	0.16	
Pulmonary trunk diameter SA (mm)	M	3.67 ± 0.43	4.55	2.99	3.92	3.42	0.14	**0.046 5**
	F	3.24 ± 0.46	4.02	2.54	3.51	2.97	0.15	
Pulmonary trunk diameter LA (mm)	M	3.22 ± 0.45	4.09	2.51	3.59	2.74	0.14	**0.019 8**
	F	2.67 ± 0.52	3.54	1.94	2.97	2.37	0.16	

From the right transarterial SA view, the two aortic arches and pulmonary trunk diameters were examined and measured. The maximum diameters of the right aortic arch and the pulmonary trunk were measured from the long axis transarterial section. Bold values denote statistical significance

CL = confidence interval; DIAM = diameter; F = female; LA = long axis; M = male; SA = short axis; SEM = standard error of the mean

**Table 3:** Measurements of the thickness of the wall of the *cavum arteriosum* (Cav. A), of the interventricular (IV) septum, of the *cavum pulmonale* (Cav. P) at the end of the diastole, and total length of the ventricle (V) obtained from a ventral approach with the long axis (LA) view of the V and right atrium

Measurement	Sex	Mean	97.5 percentile	2.5 percentile	Upper 90% CL of mean	Lower 90% CL of mean	Std. error of mean	*P*-value
IV septum (mm)	M	1.87 (1.73–2.15)	2.78	1.47	2.30	1.65	0.12	0.403 2
	F	2.23 ± 0.63	3.19	1.34	2.59	1.86	1.99	
*Cavum pulmonale* (mm)	M	1.89 ± 0.38	2.30	1.05	2.11	1.67	0.12	0.560 6
	F	2.01 ± 0.53	2.83	1.34	2.32	1.70	0.17	
*Cavum arteriosum* (mm)	M	1.66 ± 0.35	2.30	1.05	1.86	1.45	0.11	0.903 1
	F	1.63 ± 0.47	2.41	0.90	1.91	1.36	0.15	
Length ventricle (mm)	M	26.44 ± 2.48	28.61	21.35	27.88	25.00	0.78	0.266 0
	F	25.26 ± 2.10	29.07	22.26	26.48	24.05	0.66	

The thicknesses of the Cav. V and Cav. P were measured from the endocardium to the epicardium, at the point of maximum width

CL = confidence interval; F = female; M = male; SEM = standard error of the mean

**Table 4:** The pulmonary and the aortic flow velocities were measured by positioning the pulsed wave Doppler at the emergence of the pulmonary trunk and the aortic arch from a ventral approach, and the maximum peak velocities were recorded

Measurement	Sex	Mean and median	97.5 percentile	2.5 percentile	Upper 90% CL of median	Lower 90% CL of median	Std. error of mean	*P*-value
Aorta flow (m/sec)	M	0.48 (0.33–0.52)	5.50	0.19	0.60	0.30	0.51	0.811 5
	F	0.48 ± 0.15	0.71	0.24	0.57	0.39	0.04	
Pulmonary trunk flow (m/sec)	M	0.41 ± 0.16	0.60	0.16	0.50	0.31	0.05	0.222 0
	F	0.49 (0.39–0.66)	1.00	0.32	0.84	0.37	0.07	

Due to the small size of the aortas, differentiation between the right and left flows was impossible, and the flow was recorded as a single value

CL = confidence interval; F = female; M = male; SEM = standard error of the mean

## DISCUSSION

Ventral and intercostal left and right approaches for echocardiography were described by Schilliger et al. (2006). We used the same approaches in our trial, and the ventral approach proved to be the most useful in the measurement of the ventricular structures and aortic and pulmonary flows, while the lateral approach provided better visualisation of the LA, the aortas and pulmonary trunk. A ventral, SA, and transarterial section, allowed us to record the pulmonary and aortic flow with good accuracy. Normal values of intraventricular structures and cardiac flows in colubrid snakes are scarce in the literature. According to Silverman et al. (2016), the pulmonary artery flow was 0.64 m/s (± 0.11), and 0.57 (± 0.09) in male and female *Pogona vitticeps*, respectively; these values are similar to ours regarding the pulmonary trunk: 0.47 m/s (± 0.20). Poser et al. (2011) described the total ventricular outflow during the systolic peak in *Trachemys scripta elegans,* whose mean value was 0.52 m/s (± 0.07).

The echocardiography did not demonstrate any size difference in the most important cardiac structures between males and females, which is interesting due to the disproportion in the body size between males and females (Tables 2 and 3). Moreover, the pulmonary trunk diameter was bigger in the males compared to the females in both the long axis (*P* = 0.046 5) and short axis (*P* = 0.019 8). The data might suggest an increased aerobic performance in males compared to females. In *Crotalus cerastes*, males travel longer distances during the reproductive season to increase their mating opportunities (Secor 1994; Shine et al. 2003; Bonnet et al. 2005). Lima-Santos et al. (2021) described how sexual differences affect the locomotor performance and metabolism in *Tomodon dorsatus,* being faster in males and showing increased basal metabolism. The reproductive ecology of *Heterodon nasicus* has yet to be fully elucidated upon, however, male territorialism and multiple male courtships toward a single female have been described (Hoaglund and Smith 2012). Hence, a higher reproductive activity of the male and consequent sexual selection toward a higher aerobic performance can be hypothesised. Sexual selection influences animal performances when this trait directly favours an individual’s reproductive success, such as pursuing partners, increasing the area of the territory, or mating behaviours (Irschick et al. 2005; Husak et al. 2006; Husak et al. 2008).

Congenital defects, cardiomyopathies, valvulopathies, and pericardial diseases are known to occur in ophidians and other reptiles (Schilliger and Girling 2019). An echocardiography is a suitable method for the anatomical and clinical investigation of cardiac structures in snakes. Standardisation of cardiac parameters in terms of the flow, and size of the major structures can improve the diagnostic value of echocardiography, allowing the early diagnosis of cardiac illnesses even before the appearance of clinical signs. Further studies on the cardiology of snakes, aimed at the creation of a database regarding echocardiography, electrocardiogram, and echo-Doppler are strongly encouraged. An important limitation of our work was the small number of subjects involved, which hindered the statistical analysis. However, to the knowledge of the authors, this is the first study aimed to describe cardiac structures and flows in *Heterodon nasicus.*
